# The efficacy of low-level laser therapy in alleviating genitourinary syndrome of menopause and stress urinary incontinence: the pilot study

**DOI:** 10.3389/fmed.2025.1574646

**Published:** 2025-05-23

**Authors:** Pei-Chi Wu, Ho-Hsiung Lin, Chi-Hau Chen, Yih-Shing Kuo

**Affiliations:** ^1^Department of Obstetrics and Gynecology, National Taiwan University Hospital, Taipei City, Taiwan; ^2^Department of Obstetrics and Gynecology, National Taiwan University Hospital Bei-Hu Branch, Taipei City, Taiwan; ^3^Department of Obstetrics and Gynecology, Far Eastern Memorial Hospital, Taipei City, Taiwan

**Keywords:** genitourinary syndrome of menopause, stress urinary incontinence, low-level laser therapy, lower urinary tract symptoms, female sexual function

## Abstract

**Objective:**

Genitourinary syndrome of menopause (GSM) and stress urinary incontinence (SUI) are prevalent among women. While low-level laser therapy (LLLT) has been employed for wound healing, no studies have explored its effectiveness in treating GSM and SUI.

**Methods:**

Between September 2022 and August 2023, all women received LLLT for GSM and SUI at the gynecologic outpatient clinic of a hospital were retrospectively reviewed. The treatment was administered once a week for 8 weeks. Vaginal Health Index (VHI) and pre- and post-treatment questionnaires were utilized to assess lower urinary tract symptoms (LUTS).

**Results:**

A total of 41 women were enrolled, including 24 (59%) with SUI and 22 (52%) with GSM (5 women reported both conditions). Significant improvements were observed in all VHI items for both groups. Total scores for ICIQ-SF (International Consultation on Incontinence Questionnaire-Short Form), UDI-6 (Urogenital Distress Inventory-6), IIQ-7(Incontinence Impact Questionnaire-7), USS (Urgency Severity Scale), and OABSS (Overactive Bladder Symptom Score) demonstrated significant enhancements in all women and specifically in the GSM group. In the SUI group, USS and OABSS significantly improved, with more domains in KHQ (King’s Health Questionnaire) showing improvement compared to the GSM group. In the GSM group, lubrication, pain, and the total score of FSFI (Female Sexual Function Index) improved significantly, while no significant enhancement in sexual function was observed in the SUI group.

**Conclusion:**

This pilot study indicates that LLLT is a safe, cost-effective, and straightforward treatment for alleviating GSM and SUI symptoms. Women with GSM experienced improvements in LUTS and sexual function, while women with SUI demonstrated improvements in urinary urgency and LUTS-related quality of life. This study represents the first application of LLLT in gynecology.

## 1 Introduction

Genitourinary syndrome of menopause (GSM) encompasses a spectrum of distressing symptoms, including vaginal dryness and associated issues such as dyspareunia, vulvar itching, urinary urgency, and recurrent vaginal and urinary tract infections (1), affecting up to 84% of postmenopausal women (2). These symptoms significantly impact the quality of life for affected individuals. Conventional treatments, such as hormone replacement therapy and topical estrogen creams, come with contraindications and may not yield satisfactory outcomes for everyone (1). Therefore, there exists a compelling need to explore alternative therapeutic avenues.

Stress urinary incontinence (SUI) is defined as the involuntary loss of urine during physical exertion, including sports activities, or during sneezing or coughing (3), affecting about half of postmenopausal women in Asian population (4). Although it is a component of lower urinary tract symptoms, the primary etiology of SUI is the loss of paraurethral support, which is not solely attributable to age-related vaginal atrophy.

Vaginal laser therapy has emerged as one such alternative treatment modality, garnering attention over the past decade. The mechanism involves stimulating collagen production and improving blood flow in the pelvic floor, thereby restoring vaginal health (5). Devices on the market can be categorized as ablative or non-ablative lasers, both aimed at promoting regeneration and rejuvenation of the vaginal mucosa and endopelvic fascia (6). Consequently, the use of vaginal laser therapy for both GSM and SUI has steadily gained popularity.

Low-level laser therapy (LLLT), also known as cold laser therapy or photobiomodulation (7), offers a non-invasive treatment option. The treatment mechanism involves the absorption of light energy by cells, initiating a series of biochemical reactions that enhance cellular metabolism, increase blood circulation, and reduce inflammation—ultimately promoting tissue healing (7). Despite its widespread use in dermatology, orthopedics, rehabilitation, and pain management as a cost-effective therapy, the application of LLLT in the gynecological field remains largely unexplored, though it holds promising potential.

LLLT presents itself as a safe and non-invasive approach to alleviating symptoms of GSM and SUI. This study aims to assess the treatment effects of LLLT on both GSM and SUI.

## 2 Materials and methods

Between September 2022 and August 2023, the medical records of 41 women who underwent Low-Level Laser Therapy (LLLT) at the gynecologic outpatient clinic were retrospectively reviewed. Prior to any examination or treatment, written informed consent was obtained. This study was approved by the Research Ethics Committee of National Taiwan University Hospital (ID NO.202310054RIN) and was registered at ClinicalTrials.gov (NCT06136975).

The inclusion criteria for LLLT included GSM and SUI, as previously outlined in a high-energy laser study (6). Menopause was defined as the absence of spontaneous menstruation for at least 1 year. GSM was characterized by vulvovaginal dryness and associated symptoms, such as irritation, dyspareunia, or LUTS, including urinary urgency, dysuria, and recurrent urinary tract infections after menopause (1). SUI was defined as the involuntary loss of urine during physical exertion, including exercise, or during sneezing or coughing (8). The exclusion criteria included stage 2 or higher pelvic organ prolapse according to the Pelvic Organ Prolapse-Quantification System (9), ongoing urinary tract infection or vaginitis with a pathogen within 2 weeks prior to the first clinical interview, bladder calculus, neurogenic bladder resulting from radical hysterectomy or central nervous system injury, and preexisting malignant pelvic tumors.

Chart reviews included comprehensive assessments of patients’ medical histories. Participants underwent pelvic examinations both before and after LLLT to evaluate vaginal health using the Vaginal Health Index (VHI) (10). Additionally, participants completed the following questionnaires before and after treatment to subjectively assess LUTS and their impact on quality of life: the International Consultation on Incontinence Questionnaire-Short Form (11); the Incontinence Impact Questionnaire-7 (IIQ-7) (12); the Urogenital Distress Inventory-6 (UDI-6) (12); the Overactive Bladder Symptoms Score (OABSS) (13); the Urgency Severity Scale (USS) (14); and the King’s Health Questionnaire (KHQ) (15). The sexual function was assessed via the Female Sexual Function Index (FSFI) (16). Post-treatment assessments were conducted immediately following the final session.

Regarding the LLLT protocol, the treatment was administered once a week for 8 weeks. Patients were placed in the supine position for the procedure, and the laser, with a Gallium-Aluminum-Arsenide gain medium, was introduced into the vagina using a silicone vaginal probe inserted by a physician for 30 min (wavelength 660 nm, power density 18.17 mW/cm^2^, energy density 0.018 J/cm^2^s, total energy density 32.4 J/cm^2^). Post-treatment education included abstaining from sexual activity and baths/hot springs for 2 days and refraining from vaginal douching. Some participants chose to conclude the treatment course at the 4th week due to satisfactory treatment effects.

Statistical analyses were conducted using MedCalc Statistical Software version 18.10.2 (MedCalc Software bvba, Ostend, Belgium). Between-group comparisons utilized independent *t*-tests and Mann-Whitney U tests. Before-and-after treatment comparisons employed Wilcoxon-signed rank tests and paired *t*-tests. A *p*-value less than 0.05 was considered statistically significant.

## 3 Results

A total of 41 women were included in the final analysis, including 24 (59%) with SUI, 22 (52%) with GSM, and 5 who reported both conditions. Eleven women chose to undergo only four sessions of LLLT due to satisfactory outcomes achieved early in the treatment course. The SUI group was significantly younger than the GSM group (56.7 ± 11.5 years vs. 59.5 ± 7.0 years, *p* = 0.024).

Objective assessment of vaginal health between groups and pre- and post- treatment, as measured by the Vaginal Health Index (VHI), are presented in Table 1. All VHI items showed significant improvement in both SUI and GSM groups. Although vaginal health differed significantly between the SUI and GSM groups prior to LLLT, similar health levels were observed following treatment. The total VHI scores exceeded 15, which is the cutoff value for adequate vaginal health, in both groups (SUI vs GSM = 20.1 ± 2.6 vs. 19.9 ± 3.8, *p* = 0.157).

**TABLE 1:** Baseline characteristics and vaginal health index (VHI) of women received low-energy laser therapy (LLLT) for genitourinary syndrome of menopause (GSM) or stress urinary incontinence (SUI) (*n* = 41).

Variables	All (*n* = 41)			Women with SUI (*n* = 24)			Women with GSM (*n* = 22)			*p* ^c^	
Age (years)	57.9 ± 9.4			56.7 ± 11.5			59.5 ± 7.0			0.0240^d^	
Parity ^a^	2 (1–3)			2 (1–3)			2 (1–3)			0.4826	
	Before LLLT	After LLLT	*p* ^b^	Before LLLT	After LLLT	*p* ^b^	Before LLLT	After LLLT	*p* ^b^	Before LLLT	After LLLT
**VHI**											
Elasticity	2.8 ± 0.8	4.0 ± 0.8	0.0001^d^	3.0 ± 0.9	4.1 ± 0.7	0.0025^d^	2.5 ± 0.7	3.9 ± 0.8	0.0016^d^	0.0406^d^	0.6813
Fluid	2.4 ± 1.4	4.1 ± 0.8	<0.0001^d^	2.6 ± 1.6	4.2 ± 0.6	0.0028^d^	2.0 ± 1.1	4.0 ± 0.9	0.0004^d^	0.1835	0.7574
pH	1.8 ± 1.0	3.2 ± 1.2	0.0002^d^	2.1 ± 1.0	3.4 ± 1.0	0.0187^d^	1.4 ± 0.6	3.2 ± 1.3	0.0004^d^	0.0094^d^	0.8404
Integrity	3.2 ± 1.0	4.5 ± 0.6	<0.0001^d^	3.5 ± 1.1	4.4 ± 0.6	0.0111^d^	3.0 ± 0.8	4.5 ± 0.7	0.0003^d^	0.0281^d^	0.3629
Moisture	2.5 ± 1.2	4.2 ± 1.0	<0.0001^d^	2.9 ± 1.4	4.1 ± 0.9	0.0046^d^	2.0 ± 0.7	4.2 ± 1.0	0.0001^d^	0.0383^d^	0.4242
Total score	12.8 ± 4.5	20.1 ± 3.4	<0.0001^d^	14.1 ± 5.1	20.1 ± 2.6	0.0001^d^	10.9 ± 2.9	19.9 ± 3.8	<0.0001^d^	0.0090^d^	0.1570

Data presented as mean ± standard deviation, or number (percentage). SUI, stress urinary incontinence; GSM, genitourinary syndrome of menopause; LLLT, low-level laser therapy; VHI, vaginal health index.

*^a^*Data presented as number (range).

*^b^*Difference between pre- and post-treatment was analyzed by Wilcoxon signed-rank or paired-*t* test.

*^c^*Difference between subgroups was analyzed by Mann-Whitney U or independent *t* test.

*^d^*Statistically significant difference.

Table 2 presents results from subjective questionnaires evaluating LUTS and quality of life before and after treatment. Significant improvements were noted in the total scores of ICIQ-SF, UDI-6, IIQ-7, USS, and OABSS after LLLT among all women, particularly in the GSM group. In the SUI group, only USS and OABSS showed significant improvement after LLLT. Significant between-group differences in ICIQ-SF scores were observed both before (SUI vs. GSM = 9.5 ± 5.1 vs. 4.8 ± 4.4, *p* = 0.0019) and after LLLT (SUI vs. GSM = 6.4 ± 5.3 vs. 2.9 ± 4.4, *p* = 0.0143). Improvements were present, though some did not reach statistical significance. Conversely, more domains in the KHQ showed post-treatment significant improvement in the SUI group than in the GSM group. In the SUI group, domains including general health perception, incontinence impact, role limitations, physical limitations, and symptom severity all significantly improved after LLLT. In contrast, only general health perception and symptom severity significantly improved in the GSM group.

**TABLE 2:** Comparisons of questionnaires regarding lower urinary tract symptoms (LUTS) and quality of life of women received low-energy laser therapy (LLLT) for genitourinary syndrome of menopause (GSM) or stress urinary incontinence (SUI) (*n* = 41).

Variables	All (*n* = 41)			Women with SUI (*n* = 24)			Women with GSM (*n* = 22)			*p* ^b^	
	Before LLLT	After LLLT	*p* ^a^	Before LLLT	After LLLT	*p* ^a^	Before LLLT	After LLLT	*p* ^a^	Before LLLT	After LLLT
**LUTS**											
ICIQ-SF	6.5 ± 5.3	4.1 ± 4.8	0.0122^c^	9.5 ± 5.1	6.4 ± 5.3	0.0512	4.8 ± 4.4	2.9 ± 4.4	0.0179^c^	0.0019^c^	0.0143^c^
UDI-6	5.0 ± 3.3	3.6 ± 2.9	0.0085^c^	5.7 ± 3.3	3.9 ± 3.2	0.0733	5.0 ± 3.2	3.4 ± 2.7	0.0103^c^	0.3620	0.7900
IIQ-7	4.9 ± 0.9	2.7 ± 4.5	0.0019^c^	5.6 ± 5.3	3.4 ± 5.6	0.0831	4.6 ± 5.5	1.7 ± 2.5	0.0021^c^	0.3199	0.3969
USS	1.3 ± 0.9	1.0 ± 0.9	0.0006^c^	1.3 ± 0.9	0.9 ± 1.0	0.0083^c^	1.5 ± 1.1	0.9 ± 0.8	0.0067^c^	0.6670	0.8541
OABSS	4.5 ± 3.2	3.1 ± 2.9	0.0016^c^	4.8 ± 3.5	3.2 ± 3.3	0.0235^c^	4.5 ± 3.3	2.9 ± 2.4	0.0078^c^	0.8393	1.0000
**KHQ**											
General health perception	36.4 ± 25.3	22.9 ± 16.3	0.0031^c^	33.8 ± 24.7	12.5 ± 13.2	0.0244^c^	33.8 ± 28.4	26.5 ± 16.5	0.0197^c^	0.6348	0.0353^c^
Incontinence impact	33.3 ± 32.3	17.9 ± 23.5	0.0383^c^	40.0 ± 33.5	18.2 ± 27.3	0.0107^c^	26.7 ± 31.7	17.5 ± 23.2	0.3981	0.1572	0.9215
Role limitation	23.3 ± 28.4	16.7 ± 23.6	0.1646	27.0 ± 33.0	15.2 ± 20.4	0.0340^c^	19.2 ± 26.6	16.7 ± 24.2	0.9438	0.4100	0.9264
Physical limitations	31.0 ± 26.9	17.3 ± 18.9	0.0355^c^	38.3 ± 29.2	21.7 ± 20.9	0.0355^c^	23.3 ± 22.6	14.9 ± 17.5	0.1065	0.0942	0.4046
Social limitations	17.8 ± 22.3	10.3 ± 19.2	0.3178	16.1 ± 24.6	10.2 ± 16.7	0.2334	11.9 ± 18.2	8.2 ± 19.5	0.4422	0.8004	0.3235
Personal relationships	10.0 ± 22.2	4.9 ± 14.5	0.1634	14.2 ± 27.2	8.3 ± 19.5	0.3340	8.3 ± 18.3	5.3 ± 16.7	0.1635	0.4755	0.3420
Emotions	27.9 ± 32.5	12.9 ± 18.3	0.0180^c^	31.2 ± 31.1	16.2 ± 16.0	0.1366	20.0 ± 30.7	10.5 ± 18.5	0.0581	0.0647	0.1506
Sleep/energy	27.1 ± 25.6	17.9 ± 16.9	0.1331	26.7 ± 26.2	12.5 ± 14.4	0.1255	25.8 ± 24.5	22.2 ± 17.1	0.5235	0.9888	0.1237
Severity measures	35.2 ± 29.6	18.3 ± 23.5	0.0012^c^	49.2 ± 29.0	26.4 ± 23.0	0.0093^c^	22.1 ± 21.7	13.9 ± 22.7	0.0053^c^	0.0028^c^	0.0508

Data presented as mean ± standard deviation, or number (percentage). SUI, stress urinary incontinence; GSM, genitourinary syndrome of menopause; LLLT, low-level laser therapy; LUTS, lower urinary tract symptoms; ICIQ-SF, International Consultation on Incontinence Questionnaire - Short Form; UDI-6, Urogenital Distress Inventory-6; IIQ-7, Incontinence Impact Questionnaire-7; USS, Urgency Severity Scale; OABSS, Overactive Bladder Symptom Score; KHQ, King’s Health Questionnaire.

*^a^*Difference between pre- and post-treatment was analyzed by Wilcoxon signed-rank or paired-*t* test.

*^b^*Difference between subgroups was analyzed by Mann-Whitney U or independent *t* test.

*^c^*Statistically significant difference.

Table 3 presents the Female Sexual Function Index (FSFI) scores. Significant improvements in lubrication were observed in both the overall cohort and the GSM group following LLLT. Pain and the total FSFI score were significantly improved in the GSM group. However, none of the FSFI items revealed statistically significant differences in the SUI group.

**TABLE 3:** Comparisons of questionnaire regarding female sexual function of women received low-energy laser therapy (LLLT) for genitourinary syndrome of menopause (GSM) or stress urinary incontinence (SUI) (*n* = 41).

Variables	All (*n* = 41)			Women with SUI (*n* = 24)			Women with GSM (*n* = 22)			*p* ^b^	
	Before LLLT	After LLLT	*p* ^a^	Before LLLT	After LLLT	*p* ^a^	Before LLLT	After LLLT	*p* ^a^	Before LLLT	After LLLT
**FSFI**											
Desire	3.5 ± 1.5	4.0 ± 2.5	0.8415	3.6 ± 1.5	4.1 ± 3.2	0.5873	3.4 ± 1.4	3.6 ± 1.8	0.3183	0.4073	0.9733
Arousal	6.9 ± 5.2	7.4 ± 5.6	0.6039	7.4 ± 5.6	6.0 ± 5.2	0.2587	6.3 ± 5.0	7.1 ± 6.2	0.5183	0.4857	0.5183
Lubrication	6.9 ± 6.2	8.7 ± 6.7	0.0136^c^	7.6 ± 6.8	6.6 ± 6.7	0.8646	5.8 ± 5.4	8.9 ± 7.2	0.0119^c^	0.3664	0.3231
Orgasm	5.5 ± 4.8	6.4 ± 5.6	0.0643	5.6 ± 5.2	5.2 ± 5.7	0.5176	5.3 ± 4.7	6.3 ± 5.7	0.0602	0.8968	0.4995
Pain	6.0 ± 5.3	7.7 ± 5.6	0.0696	6.9 ± 6.4	7.6 ± 5.7	0.5002	5.5 ± 4.4	7.7 ± 5.7	0.0278^c^	0.5286	0.9475
Satisfaction	7.1 ± 4.8	8.1 ± 6.3	0.3152	7.5 ± 4.6	7.2 ± 7.0	0.8655	7.0 ± 4.7	7.5 ± 6.3	0.4505	0.6832	0.7401
Total	13.4 ± 9.0	15.8 ± 10.2	0.1301	14.0 ± 9.8	14.3 ± 10.3	0.9889	12.3 ± 8.2	15.1 ± 10.9	0.0300^c^	0.5244	0.9222

Data presented as mean ± standard deviation, or number (percentage). SUI: stress urinary incontinence; GSM: genitourinary syndrome of menopause; LLLT: low-level laser therapy; FSFI: female sexual function index.

*^a^*Difference between pre- and post-treatment was analyzed by Wilcoxon signed-rank or paired-*t* test.

*^b^*Difference between subgroups was analyzed by Mann-Whitney U or independent *t* test.

*^c^*Statistically significant difference.

## 4 Discussion

In our study, we observed significant improvements in GSM, reflected not only in subjective assessments of LUTS and Female Sexual Function but also in objective findings, as evidenced by improvements in the VHI following LLLT. Previous studies have investigated traditional laser acupuncture, skin-adhesive patches, and general external irradiation for the treatment of SUI, OAB, and vestibulodynia (17–19). To the best of our knowledge, our study is the first to employ a vaginal probe for LLLT targeting both GSM and SUI.

Over the past two decades, the utilization of vaginal laser therapy has seen a steady rise, predominantly with ablative CO_2_ lasers and non-ablative Er:YAG lasers (5, 6). As a non-hormonal therapy, vaginal laser improves GSM and SUI by enhancing collagen production and promoting tissue remodeling, ultimately increasing the thickness of the vaginal epithelium. Recent systematic reviews have shown significantly positive short-term effects of both high-energy lasers for GSM and SUI (20, 21) without major complication. However, in our daily practice, the most commonly reported minor adverse effect of high-energy lasers is heat and tenderness during the procedure. Additionally, the need to rotate the probe without jelly to avoid a barrier between the laser beam and vaginal mucosa can cause rubbing pain, especially for severe GSM patients. While smaller probes are available, their efficacy may decrease if not appropriately fitted to the vagina.

LLLT has found wide application in various medical fields since its first experiment on hair regrowth more than 50 years ago (22, 23). The molecular effect of LLLT, known as photobiomodulation (7), includes the excitation of the respiratory chain in mitochondria, leading to increased ATP production and cellular activity. Moreover, LLLT alters mitochondrial retrograde signaling, enhancing cell proliferation (24). Prior to our study, LLLT had been extensively applied to improve skin and mucosal wound healing, (25, 26) increased osteoblast proliferation, (27) and reduce inflammatory pain, (28) with various energy and power densities.

Genitourinary tissues undergo atrophy after menopause, causing inflammation, pain, and LUTS. While hormonal therapy remains the first-line treatment with demonstrated efficacy for GSM, it may not be suitable for certain patients, such as those with breast cancer or gynecological malignancies. In this aging era, elderly individuals may also struggle to apply estrogen cream by themselves. As a non-invasive treatment, LLLT could offer significant assistance to those with GSM.

The mechanisms for treating atrophy-related symptoms are similar: improving cellular metabolism, increasing microcirculation, reducing focal inflammation, and promoting tissue healing, all of which have been studied and proven in previous LLLT studies. Efficacy and safety for transmucosal and transdermal applications have been investigated in fragile patients, such as those with head and neck cancer, even with commercially available devices for home use. The application of LLLT to chronic vaginal inflammation, as seen in GSM, is both novel and rational.

Compared to chronic wounds, vaginal epithelium is usually intact in women with GSM, making this application even safer than its current use in dermatology. The treatment courses were comfortable and uneventful, with no reported pain, heat, or acute wounds during or after treatment, which contrasts with observations during traditional high-energy vaginal laser procedures (29). Additionally, no incidents of vaginal or urinary tract infections were reported post-treatment. In summary, there were no noticeable adverse effects during the treatment course, a crucial consideration for the elderly population.

Addressing the treatment effects, our study reported significant improvements in both objective vaginal health and subjective quality of life. Given that the molecular effect of LLLT stimulates cellular metabolism and enhances cellular function, it is reasonable to expect stimulation of the vaginal epithelium, resulting in increased mucus formation, lubrication, and reduction of inflammation and pain. All domains in the VHI showed significant improvement after LLLT, in both SUI and GSM groups. Intriguingly, these improvements were less pronounced in premenopausal women. We postulate that once the atrophic epithelium heals and recovers, improvements in elasticity, fluid volume, moisture, and pH value may occur, creating a more favorable microenvironment in the vagina. Further histological studies would be valuable for confirmation. The degree of atrophy in the epithelium may correlate with the extent of improvement observed. Table 1 shows significant differences in VHI between the two groups before treatment, indicating better vaginal health in the SUI group. However, both groups reached a similar health level (VHI total score > 15) after LLLT. Follow-up assessments will help determine if vaginitis decreases over time.

Not only the vagina but also the mucosa in the lower urinary tract undergo atrophy and dysfunction after menopause, leading to various LUTS. Women with SUI were included as one of the indications for LLLT, mirroring high-energy laser applications. While objective improvements in VHI were statistically significant for all VHI items in both groups, the treatment effects on LUTS were not significant in women with SUI only. The mechanism of SUI primarily involves structural aspects, and the insignificant improvement may stem from our application of relatively low energy and low power density settings, compared to those used for wound healing and promoting osteoblast function. If a woman had both GSM and LUTS simultaneously, these symptoms could be relieved by the same mechanism. However, in women with SUI only, the energy setting may not improve the endurance of the fascia and urethral support. Nevertheless, we observed significant improvement in USS and OABSS in the SUI group. Women may experience a combination of complex LUTS simultaneously. Given that storage symptoms of bladder function are closely linked to neuromuscular aspects (30, 31), it is plausible to anticipate the neuromodulatory effects of LLLT on urinary urgency and Overactive Bladder symptoms (OAB). Consequently, significant enhancements in various domains of the KHQ within the SUI groups are evident for this reason. These improvements appear to be symptom-specific rather than generalized, signifying notable progress in areas that hold significance for the patients. To delve deeper into this aspect, we plan to conduct a subsequent energy-density based study focusing on SUI and OAB.

As a consequence of improved vaginal health, dryness and pain were significantly reduced. Pain and lubrication domains in the FSFI also improved in GSM patients, along with improvements in the total score. However, the total score of FSFI did not reach an ideal level (more than 26) after LLLT, and the improvement was not significant in the analysis of all women. Apart from the small number of sexually active women in our data, we hypothesize that the observed time for the improvement of pain and lubrication might not be long enough. Theoretically, increased desire and arousal may follow reassurance of pain relief and adequate lubrication, leading to subsequent satisfaction and an increase in the total FSFI score. Hence, achieving a comprehensive improvement in all domains after treatment requires time. Pain relief is identified as a crucial first step in enhancing sexual function.

The retrospective design of this study and the absence of a control are limitations; however, the significant treatment effects observed in this pilot study, particularly in a specific population, provide valuable insights for patients and caregivers. These improvements in symptoms are specific to the original symptoms of the patients, reflecting the authenticity of our data. This study introduces a novel, safe, and straightforward application for LLLT. For women with GSM unsuitable for traditional medication, LLLT emerges as a comfortable, cost-effective, and highly efficacious treatment choice. The cost per session to patients for LLLT is approximately one-fifth that of high-energy vaginal laser therapy, and the device cost to healthcare providers is also roughly one-fifth. Although a formal cost analysis was not conducted, the relatively lower equipment and treatment costs associated with LLLT compared to high-energy laser systems suggest it may be a more affordable option for both patients and healthcare providers. This study represents the pioneering application of LLLT in the field of gynecology. Further prospective randomized controlled trials and histological studies are essential to confirm the therapeutic effects of LLLT. Future research directions may include a prospective study aimed at optimizing LLLT dosing and evaluating its long-term efficacy, histological assessment of vaginal mucosal changes following LLLT, and investigation of intravesical LLLT for the treatment of urotheliogenic overactive bladder.

## 5 Conclusion

Low-level laser therapy appears to be a safe, comfortable, low-cost, and easily administered treatment, with potential benefits in improving vaginal health among women with GSM and SUI. Improvements in LUTS and sexual function were observed in the GSM group, and the SUI group showed enhanced urinary urgency and LUTS-related quality of life. While these preliminary findings are promising, they should be interpreted with caution due to the retrospective design, lack of a control group, and limited sample size. Further prospective controlled studies are needed to confirm these results and to better define the therapeutic role of LLLT in this population.

## Data Availability

The raw data supporting the conclusions of this article will be made available by the authors, without undue reservation.

## References

[1] Phillips N Bachmann G. The genitourinary syndrome of menopause. *Menopause.* (2021) 28:579–88. 10.1097/GME.0000000000001728 33534428

[2] Sarmento A Costa A Vieira-Baptista P Giraldo P Eleutério J Jr. Gonçalves A. Genitourinary syndrome of menopause: Epidemiology, physiopathology, clinical manifestation and diagnostic. *Front Reprod Health.* (2021) 3:779398. 10.3389/frph.2021.779398 36304000 PMC9580828

[3] Haylen B de Ridder D Freeman R Swift S Berghmans B Lee J An International Urogynecological Association (IUGA)/International Continence Society (ICS) joint report on the terminology for female pelvic floor dysfunction. *Neurourol Urodyn.* (2010) 29:4–20. 10.1002/nau.20798 19941278

[4] Huang H Ding G Li M Deng Y Cheng Y Jin H. Menopause and stress urinary incontinence: The risk factors of stress urinary incontinence in perimenopausal and postmenopausal women. *J Obstet Gynaecol Res*. (2023) 49:2509–18. 10.1111/jog.15742 37443520

[5] Gambacciani M Palacios S. Laser therapy for the restoration of vaginal function. *Maturitas.* (2017) 99:10–5. 10.1016/j.maturitas.2017.01.012 28364861

[6] Long C Wu P Chen H Lin KL Loo Z Liu Y Changes in sexual function and vaginal topography using transperineal ultrasound after vaginal laser treatment for women with stress urinary incontinence. *Sci Rep.* (2022) 12:3435. 10.1038/s41598-022-06601-0 35236871 PMC8891315

[7] Rola P Doroszko A Derkacz A. The use of low-level energy laser radiation in basic and clinical research. *Adv Clin Exp Med.* (2014) 23:835–42. 10.17219/acem/37263 25491701

[8] Haylen B Maher C Barber M Camargo S Dandolu V Digesu A An International urogynecological association (IUGA)/International Continence Society (ICS) joint report on the terminology for female pelvic organ prolapse (POP). *Int Urogynecol J.* (2016) 27:165–94. 10.1007/s00192-015-2932-1 26755051

[9] Bump R Mattiasson A Bo K Brubaker L DeLancey J Klarskov P The standardization of terminology of female pelvic organ prolapse and pelvic floor dysfunction. *Am J Obstet Gynecol.* (1996) 175:10–7. 10.1016/s0002-9378(96)70243-0 8694033

[10] Palacios S. Assessing symptomatic vulvar, vaginal, and lower urinary tract atrophy. *Climacteric.* (2019) 22:348–51. 10.1080/13697137.2019.1600499 31157569

[11] Huang L Zhang S Wu S Ma L Deng X. The Chinese version of ICIQ: A useful tool in clinical practice and research on urinary incontinence. *Neurourol Urodyn.* (2008) 27:522–4. 10.1002/nau.20546 18351586

[12] Uebersax J Wyman J Shumaker S McClish D Fantl J. Short forms to assess life quality and symptom distress for urinary incontinence in women: The Incontinence impact questionnaire and the urogenital distress inventory. continence program for women research group. *Neurourol Urodyn.* (1995) 14:131–9. 10.1002/nau.1930140206 7780440

[13] Homma Y Yoshida M Seki N Yokoyama O Kakizaki H Gotoh M Symptom assessment tool for overactive bladder syndrome–overactive bladder symptom score. *Urology.* (2006) 68:318–23. 10.1016/j.urology.2006.02.042 16904444

[14] Nixon A Colman S Sabounjian L Sandage B Schwiderski U Staskin D A validated patient reported measure of urinary urgency severity in overactive bladder for use in clinical trials. *J Urol.* (2005) 174:604–7. 10.1097/01.ju.0000165461.38088.7b 16006914

[15] Kelleher C Cardozo L Khullar V Salavatore S. A new questionnaire to assess the quality of life of urinary incontinent women. *Br J Obstet Gynaecol.* (1997) 104:1374–9. 10.1111/j.1471-0528.1997.tb11006.x 9422015

[16] Liu H Yu J Chen Y He P Zhou L Tang X Sexual function in cervical cancer patients: Psychometric properties and performance of a Chinese version of the Female Sexual Function Index. *Eur J Oncol Nurs.* (2016) 20:24–30. 10.1016/j.ejon.2015.06.007 26160725

[17] Mousa G Yousef A El-Sayed R El-Mekawy E El Deen Abdel A Ahmed Hamada H Effect of low level laser on pelvic floor muscles and fascia in cases of stress urinary incontinence: A randomized controlled trial. *Physiother Quart.* (2021) 29:22–7. 10.5114/pq.2021.102549

[18] Hwang W Kim Y Lee S Suh D Kim K No J. Efficacy and safety of skin-adhesive low-level light therapy for overactive bladder: A phase III study. *Int Urogynecol J*. (2022) 33:3573–80. 10.1007/s00192-022-05153-1 35389054 PMC9666307

[19] Lev-Sagie A Kopitman A Brzezinski A. Low-level laser therapy for the treatment of provoked vestibulodynia-A randomized. *Placebo-Controlled Pilot Trial. J Sex Med*. (2017) 14:1403–11. 10.1016/j.jsxm.2017.09.004 28970071

[20] D’Oria O Giannini A Buzzaccarini G Tinelli A Corrado G Frega A Fractional CO2 laser for vulvo-vaginal atrophy in gynecologic cancer patients: A valid therapeutic choice? A systematic review. *Eur J Obstet Gynecol Reprod Biol.* (2022) 277:84–9. 10.1016/j.ejogrb.2022.08.012 36037664

[21] Ranjbar A Mehrnoush V Darsareh F Kotb A Zakaria A Shekari M Vaginal laser therapy for stress urinary incontinence: A systematic review of prospective randomized clinical trials. *J Menopausal Med.* (2022) 28:103–11. 10.6118/jmm.22017 36647273 PMC9843031

[22] Glass G. Photobiomodulation: The clinical applications of low-level light therapy. *Aesthet Surg J.* (2021) 41:723–38. 10.1093/asj/sjab025 33471046

[23] Mester E Szende B Gärtner P. The effect of laser beams on the growth of hair in mice. *Radiobiol Radiother.* (1968) 9:621–6. 5732466

[24] Gao X Xing D. Molecular mechanisms of cell proliferation induced by low power laser irradiation. *J Biomed Sci.* (2009) 16:4. 10.1186/1423-0127-16-4 19272168 PMC2644974

[25] Medrado A Pugliese L Reis S Andrade A. Influence of low level laser therapy on wound healing and its biological action upon myofibroblasts. *Lasers Surg Med.* (2003) 32:239–44. 10.1002/lsm.10126 12605432

[26] Zecha J Raber-Durlacher J Nair R Epstein J Sonis S Elad S Low level laser therapy/photobiomodulation in the management of side effects of chemoradiation therapy in head and neck cancer: Part 1: Mechanisms of action, dosimetric, and safety considerations. *Support Care Cancer.* (2016) 24:2781–92. 10.1007/s00520-016-3152-z 26984240 PMC4846477

[27] Garzón J Baldion P Grajales M Escobar L. Response of osteoblastic cells to low-level laser treatment: A systematic review. *Lasers Med Sci.* (2022) 37:3031–49. 10.1007/s10103-022-03587-z 35751706

[28] Bjordal J Johnson M Iversen V Aimbire F Lopes-Martins R. Low-level laser therapy in acute pain: A systematic review of possible mechanisms of action and clinical effects in randomized placebo-controlled trials. *Photomed Laser Surg.* (2006) 24:158–68. 10.1089/pho.2006.24.158 16706694

[29] Li F Maheux-Lacroix S Deans R Nesbitt-Hawes E Budden A Nguyen K Effect of fractional carbon dioxide laser vs sham treatment on symptom severity in women with postmenopausal vaginal symptoms: A randomized clinical trial. *JAMA.* (2021) 326:1381–9. 10.1001/jama.2021.14892 34636862 PMC8511979

[30] Hsiao S Wu P Chang T Chen C Lin H. Urodynamic and bladder diary factors predict overactive bladder-wet in women: A comparison with overactive bladder-dry. *Int Neurourol J.* (2019) 23:69–74. 10.5213/inj.1836212.106 30943696 PMC6449656

[31] Wu P Hsiao S Lin H. Prevalence and predictors of nocturnal polyuria in females with overactive bladder syndrome. *World J Urol.* (2022) 40:519–27. 10.1007/s00345-021-03865-5 34762173

